# Benefits of Art Therapy in People Diagnosed With Personality Disorders: A Quantitative Survey

**DOI:** 10.3389/fpsyg.2020.00686

**Published:** 2020-04-15

**Authors:** Suzanne Haeyen, Farid Chakhssi, Susan Van Hooren

**Affiliations:** ^1^GGNet Centre of Mental Health, Apeldoorn, Netherlands; ^2^KENVAK Research Centre for the Arts Therapies, Heerlen, Netherlands; ^3^Department of the Arts & Psychomotor Therapies Education Programme, HAN University of Applied Sciences, Nijmegen, Netherlands; ^4^Department of Psychology, Health and Technology, Faculty of Behavioural, Management and Social Sciences, University of Twente, Enschede, Netherlands; ^5^Department of the Arts Therapies Education Programme, Zuyd University of Applied Sciences, Heerlen, Netherlands; ^6^Faculty of Psychology, Open University of the Netherlands, Heerlen, Netherlands

**Keywords:** art therapy, personality disorders cluster B and C, quantitative survey, treatment goals, indication, visual arts, benefits

## Abstract

Art therapy is widely used and effective in the treatment of patients diagnosed with Personality Disorders (PDs). Current psychotherapeutic approaches may benefit from this additional therapy to improve their efficacy. But what is the patient perspective upon this therapy? This study explored perceived benefits of art therapy for patients with PDs to let the valuable perspective of patients be taken into account. Using a quantitative survey study over 3 months (*N* = 528), GLM repeated measures and overall hierarchical regression analyses showed that the majority of the patients reported quite a lot of benefit from art therapy (mean 3.70 on a 5-point Likert scale), primarily in emotional and social functioning. The improvements are concentrated in specific target goals of which the five highest scoring goals affected were: expression of emotions, improved (more stable/positive) self-image, making own choices/autonomy, recognition of, insight in, and changing of personal patterns of feelings, behaviors and thoughts and dealing with own limitations and/or vulnerability. Patients made it clear that they perceived these target areas as having been affected by art therapy and said so at both moments in time, with a higher score after 3 months. The extent of the perceived benefits is highly dependent for patients on factors such as a non-judgmental attitude on the part of the therapist, feeling that they are taken seriously, being given sufficient freedom of expression but at the same time being offered sufficient structure and an adequate basis. Age, gender, and diagnosis cluster did not predict the magnitude of perceived benefits. Art therapy provides equal advantages to a broad target group, and so this form of therapy can be broadly indicated. The experienced benefits and the increase over time was primarily associated with the degree to which patients perceive that they can give meaningful expression to feelings in their artwork. This provides an indication for the extent of the benefits a person can experience and can also serve as a clear guiding principle for interventions by the art therapist.

## Introduction

Personality disorders (PDs) are enduring and inflexible patterns of cognitions, emotions, interpersonal functioning or impulse control that lead to significant distress or impairments with an impact on a broad range of personal and social situations (American Psychiatric Association, 2013; World Health Organization, 2015). Recent meta-analyses have shown that psychotherapy is effective for reducing PD pathology, with significant but small to moderate effect sizes (Stoffers et al., 2012; Budge et al., 2013; Cristea et al., 2017), while psychosocial functioning seems to remain severely impaired in individuals with PDs, and subsequently forms a substantial risk factor for relapse, occurrence or recurrence of symptoms (Zanarini et al., 2009, 2010; Keuroghlian et al., 2013; Chakhssi et al., 2019). Thus, current psychotherapeutic approaches may benefit from additional therapies that may improve upon their efficacy.

Art therapy is one of the therapies that is widely used as an additional therapy in the treatment of patients with PDs^1^. Art therapy is an experiential form of treatment that makes use of art media, creative processes, and the resulting artwork to improve a patient’s symptomatic functioning while enhancing their well-being. Art therapy is aimed at artistic self-expression and reflection of problematic feelings or themes and guided by an art therapist. These feelings or themes can be explored without being directly expressed in words [e.g., Moschini, 2005; Schweizer et al., 2009; Malchiodi, 2012; American Art Therapy Association, 2017; British Association of Art Therapists [BAAT]., 2017]. Difficulties with emotion-regulation are a central issue for people diagnosed with a PD (Dixon-Gordon et al., 2017; Haeyen, 2018). Emotion regulation refers to the processes of how people influence experienced emotions: when we have emotions, how we experience them and how we express them (Gross, 1998). The way people express emotions plays an important role in social interactions (Gross, 2002). Art therapy may help patients to recognize difficult emotions, to integrate conflicting thoughts, feelings or behaviors, and to find a more constructive way of dealing with them (Eisdell, 2005; Simon, 2005; Haeyen, 2011b, 2018, 2019). Art therapy is an integrative health profession which has roots in the social sciences, medicine and art (Czamanski-Cohen and Weihs, 2016).

Art therapy for patients with PDs has shown to be effective in a randomized controlled trial, in which art therapy was compared to wait list control showing large effects on PD pathology (*N* = 74) (Haeyen et al., 2018). Additional analyses showed that art therapy contributed equally to decreasing symptoms and improving well-being (Haeyen et al., 2017b). Also, in two pilot studies, art therapy among patients with primarily antisocial PDs was effective in provoking experiences and feelings (mental states) and promoting a healthy adult attitude to these feelings and experiences. It also improved self-esteem and social relations (Green et al., 1987; van den Broek et al., 2011). Several smaller non-comparative quatitative studies report positive results of art therapy regarding global functioning, treatment adherence (Franks and Whitaker, 2007; Eren et al., 2014), sense of solitude and self-centered isolation (Gatta et al., 2014), distress tolerance, service use (Springham et al., 2012), and expression of emotions (Haeyen, 2011a).

Despite the scarce evidence, this form of therapy is named in some national care guidelines (Landelijke Stuurgroep Multidisciplinaire Richtlijn ontwikkeling in de GGZ, 2008; Alliantie Kwaliteit in de geestelijke gezondheidszorg. [Akwa GGZ], 2017). While it seems to be related to positive experiences in the practice of professionals and service users, there has been little specific research of this form of therapy. RCTs and other effect studies are important, but they mainly look at the degree of change in complaints and coping. That is not the whole story. How do patients view the therapy? What do they think of it themselves? So if we ask them explicitly, we can map out the valuable perspective of the patients and let have their opinion taken into account. This could also help to facilitate a better indication for art therapy.

The experiences of patients with PDs are important, however, these have not been the subject of many studies. Of the few studies performed in samples of patients with PDs, patients perceived relatively more benefits of art therapy than all other therapies (Group psychotherapy, cognitive group therapy, problem solving group, body-oriented group therapy, household group, and pharmaco therapy) and were given significantly higher scores. Also, the perceived benefits of art therapy correlated significantly with the perceived overall benefits of the therapeutic program [Karterud and Pedersen, 2004 (*N* = 319)]. More recently, two qualitative studies [Haeyen et al., 2015 (*N* = 29); Haeyen et al., 2017a (*N* = 11)], found that participants experienced more insight in their problematic patterns of feelings, thoughts, and behavior, which had not been addressed before and that this process went beyond a conscious, cognitive level. However, these studies leave questions unanswered regarding the more specific benefits of art therapy for patients with PDs, which specific therapeutic goals are perceived, which factors are important for increasing the benefit of the therapy, and for whom art therapy shows the largest benefits. To the best of our knowledge, no study has explored whether PD patients’ demographic or clinical characteristics, as well as art therapy elements, is associated with their perceived benefits of art therapy.

Therefore, the object of the present study is to focus in depth on studying the personal benefits experienced by patients as a result of art therapy. If we know how patients experience art therapy, then we can investigate whether the extent to which benefits are experienced is influenced by characteristics that have to do with the therapist’s attitude or the extent to which patients felt they were able to express their feelings in the artwork. This study focuses on the questions: To what degree do patients diagnosed with a PD experience benefits from art therapy, does this contribute to perceived improved daily, emotional and/or social functioning and which treatment goals are addressed? Is the experienced benefit related to the extent to which patients can express their feelings in making artwork? This question was added because emotion-regulation problems are a key issue for people diagnosed with a PD. Furthermore, we wanted to explore if some factors or patient characteristics are predictive. Is the extent of benefits gained related to the attitude of the therapist, and what conditions he or she creates? And is art therapy more beneficial for certain persons than for others, looked at from the perspective of age, gender and diagnosis cluster?

## Materials and Methods

This study is a practice-based, quantitative survey study with two assessments covering a period of 3 months.

### Participants

The participants were patients (18+) with at least one DSM-5 PD (American Psychiatric Association, 2013), who were undergoing specialized treatment as usual, and where art therapy was offered as an additional therapy. Exclusion criteria for the current study were the inability to read or speak the Dutch language.

Data were collected from 539 patients with a PD at measurement points 1 and 2. In this group, 27.1% were diagnosed with a Cluster B personality disorder (Borderline PD *n* = 116, Narcissistic PD *n* = 2), 30.1% were diagnosed with a Cluster C PD (Avoidant PD *n* = 95, Dependent PD *n* = 13, Obsessive compulsive PD *n* = 23), and 34.5% with PD not otherwise specified. Most patients, 78.6%, were women. Age ranged from 19 to 65 (mean: 35.67, SD: 10.12). In a number of cases (*n* = 104) diagnostic information was missing. These cases were still included in the analyses because the patients were in treatment in PD specialized care units, which always requires a PD diagnosis. Finally data from 528 of the 539 patients were analyzed based on the completion of the questionnaires at both T1 and T2.

### Survey

The survey contained 8 questions and 13 statements about perceived benefits from art therapy, associated factors and patient characteristics.

#### Benefits

The possible benefits from art therapy were evaluated with a question about the extent to which patients had benefited from the treatment (item: “*Have you benefited from art therapy?*”) and three sub statements on improvement in their daily/emotional/social functioning during treatment thanks to art therapy (item example: *“My daily functioning has improved through art therapy.”* The overall question was formulated following the study of Karterud and Pedersen (2004). In this study every patient was asked “how much benefit did you gain from therapy x?” We added the three sub statements focused on daily/emotional/social functioning based on the practice based classification of treatment goals used in a top clinical institute for PDs. The question and the statements were scored on a 5-point Likert scale from “(1) none” to “(5) a lot.” The Cronbach’s alpha of this scale was 0.88, minimum 2.86, maximum 3.55, a range of 1.06, variance of 0.24 and a mean of 3.55. Test–retest reliability with average interval of 2 weeks showed a Pearson’s correlation of *r* = 0.89 for the overall question, *r* = 0.79 for the item about daily functioning, *r* = *0.79* for emotional, and *r* = 0.72 for social functioning.

Perceived benefits related to treatment goals were then examined using nine items which asked whether art therapy had contributed to a specific treatment goal such as a clearer self-image, increased self-confidence/self-esteem, or the expression of feelings/showing emotions (“Which personal areas have improved through art therapy?” Item examples: *“Clearer self-image (who I am, what I want, what I can do)”* or *“Making your own choices and self-determination”*). This selection of specific treatment goals emerged in previous research as relevant for this target group (Haeyen et al., 2015). The various treatment goals were evaluated by participants with a dichotomous scale asking participants to indicate for each goal whether or not it had been achieved in art therapy. The internal consistency of these items was 0.82 measured with Cronbach’s alpha. The test–retest reliability with average interval of 2 weeks of each item/each treatment goal varied from 0.51 to 0.70 measured with Spearman’s rho (ρ). These items were analyzed on a single item level and because we wanted to explore each goal by itself and therefore were not considered as a scale.

#### Associated Factors

The factors related to art therapy and the therapist’s attitude were examined using five independent statements: – the relationship with the therapist, in which the therapist adopted a non-judgmental attitude (item: *“The art therapist responds to me in a non-judgmental manner”*); – the feeling that patients were taken seriously (item: *“I feel that I am taken seriously in art therapy”*); – the degree of structure offered (item: *“I receive sufficient guidance to create artwork during the sessions”*); – the level of freedom offered in the therapy (item: *“I have sufficient freedom to express myself through working in art”*). These aspects were scored on a 5-point Likert scale from “(1) never true” to “(5) almost always true.” One item asked about the extent to which a person could express his or her feelings in creating artwork in the art therapy sessions, for which the same Likert scale was used (item: *“I am able to express my feelings through the process of making art”*). These items were analyzed on a single item level because we wanted to have a specific look at the content of each item. Test–retest results showed a Pearson’s correlation in the range of *r* = 0.50 to 0.85. More specifically: Non-judgmental therapist *r* = 0.60, Feel taken seriously *r* = 0.80, Sufficient freedom for expression *r* = 0.50, Sufficient guidance *r* = 0.70 and Expression of feelings in the art work *r* = *0.85*.

#### Patient Characteristics as Predictors

In order to investigate which participant reported more benefits than for others, the following aspects were examined: age (continuous), gender (male/female), and diagnosis cluster. Diagnosis cluster was divided into the subgroups of cluster B, cluster C, and PD (not otherwise specified), since these were the most frequent diagnoses within the entire group. The division in clusters was also based on meaningful clinical groups with a large enough sample to be looked at separately. These aspects were variables on a single item level.

### Procedure

The participants were recruited by 18 art therapists working in specialized departments for PD treatment at six large mental health care institutions in Netherlands in order to obtain a nationwide representative sample. All treatments were multidisciplinary, with a group form of art therapy (and to a much lesser extent, individual therapy) as additional therapy. Participants agreed to their participation by means of an informed consent form and then completed the questionnaire at the first measurement point (after at least three sessions) and at a second measurement point, after 3 months. The first and second author are working in one of these health care institutions. Since the data collection was performed in six institutions, we consider there was no conflict of interest. There seems to be no question of effects of social desirability bias because data collection was performed in six institutions and the questionnaire was handed out by the therapist or by colleagues, but in any case by persons other than the researchers.

The procedure for data handling was as follows: we prevented as much as possible missing data by collecting a large part of the survey digitally and missing data were not accepted to the completion of the questionnaire. For the other part, the survey was offered paper and pencil wise. If only very few missing data occurred in a questionnaire, the mean value of a variable was used in place of the missing data value for that same variable (mean substitution). The mean is a reasonable estimate for a randomly selected observation from a normal distribution. The sample seems large enough and power does not seem an issue.

### Data Analysis

Data were analyzed with IBM SPSS 24 (International Business Machines [IBM], 2013). Firstly, we investigated the level of *reported benefit from art therapy* (research question 1) (overall and in the case of daily, emotional and social functioning) and change over time using GLM repeated measures procedure with time as a within-subject factor. The nine items referring to specific treatment goals and whether these changed over time were examined based on chi square tests.

In order to examine the research question 2, we correlated the *associated factors* with the overall perceived benefits and three sub questions: benefits for daily, emotional, and social functioning.

**FIGURE 1:**
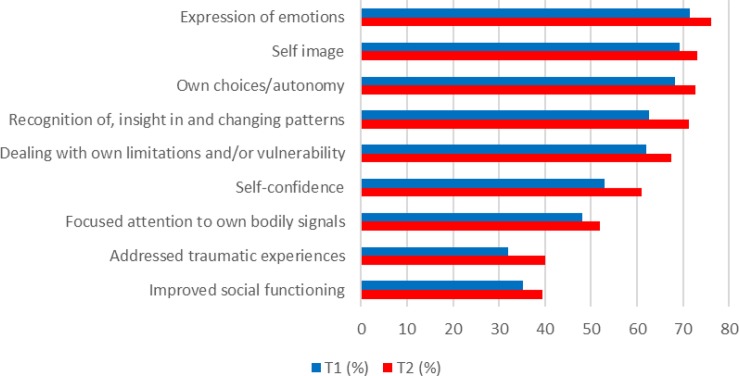
Frequencies of specified benefits of art therapy (%) (*N* = 528).

For research question 3 “to examine whether differences in patient characteristics could predict the magnitude of perceived benefits” (*patient characteristics as predictor*), we used Pearson correlations for age and chi square for gender and diagnosis. In overall hierarchical regression analyses we also tested whether benefits after 3 months could be predicted by benefits experienced at T1, the specific treatment goals, associated factors, age and gender, and PD diagnosis cluster. This model (see Figure 1) was tested for overall benefits and for the three domains, i.e., daily, emotional, and social functioning. It was tested in five steps using the enter method, i.e., step 1, perceived benefits at T1; step 2, specific treatment goals at T1; step 3, associated factors; step 4, age and gender; and step 5, cluster of PD diagnosis. This model was tested for overall benefits as well as the three domains of daily, emotional, and social functioning.

## Results

### Research Question 1: Reported Benefits of Art Therapy

The majority of patients reported that they had quite a lot of benefits from art therapy in general with a mean of 3.70 (SD = 0.99) on T2 (T1: 50.3%; T2: 56.4%). Only 2.2% (T1) and 1.5% (T2) of patients reported that they perceived no benefits in general. For benefits relating to emotional functioning, 25.2% of the patients reported these benefits at T1 and 31.8% reported this at T2. For daily and social functioning the percentages were lower (daily functioning, T1: 14.3% and T2: 16.5%; social functioning T1: 12.1% and T2: 16.9%). The results of the GLM repeated measures showed that the reported overall benefits increased after 3 months of art therapy *F*(1,527) = 13.05, *p* < 0.001. This was also the case for benefits in emotional functioning *F*(1,526) = 6.262, *p* < 0.05 and in social functioning *F*(1,527) = 5.73, *p* < 0.05, see Table 1.

**TABLE 1:** Benefits of art therapy, GLM repeated measures analyses (*N* = 528).

	T1 mean (SD)	T2 mean (SD)	Df* T1, T2	*F*	Sig.	Partial eta squared η^2^
Benefit – overall	3.56 (1.04)	3.70 (0.99)	1, 527	13.05	0.00	0.024
Daily functioning	2.57 (1)	2.57 (1.02)	1, 526	0.008	0.93	0.000
Emotional functioning	2.88 (1.06)	3.00 (1.06)	1, 526	6.262	0.01	0.012
Social functioning	2.50 (0.97)	2.60 (0.99)	1, 527	5.73	0.02	0.011

*Greenhouse–Geisser was used to adjust the degrees of freedom.

As to whether art therapy contributed to specific treatment goals, a similar pattern over time was seen, see Figure 1. At both points, the most frequently reported specific treatment goals were expression of emotions (T1: 71.5%; T2: 76.1%), improved (more stable/positive) self-image (T1: 69.2%; T2: 73.1%), making own choices/autonomy (T1: 68.3%; T2: 72.7%), recognition of, insight in and changing of personal patterns of feelings, behaviors, and thoughts (T1: 62.7%; T2: 71.2%) and dealing with own limitations and/or vulnerability (T1: 62%; T2: 67.5%). Using chi square, it was shown for each specific treatment goal that it was more often reported at T2 than at T1 (*p* < 0.01).

### Research Question 2: Associated Factors

Pearson’s correlation coefficient (*r*) was used to examine whether the reported benefits were associated with specific factors relating to the therapist’s attitude or the extent to which patients were able to experience expressing their feelings in the artwork in the art therapy sessions (see Table 2). The various aspects proved to correlate with the degree of perceived benefits. Particularly the extent to which a person can express his or her feelings in the artwork showed a high correlation (ranging from *r* = 0.47 for social functioning to *r* = 0.72 for overall benefit).

**TABLE 2:** Pearson correlation (*r*) between benefits and a number of variables at T2 (*N* = 528).

Items	Did you benefit from art therapy?	My daily functioning improved thanks to art therapy	My emotional functioning improved thanks to art therapy	My social functioning improved thanks to art therapy
Non-judgmental therapist	0.18**	0.16**	0.14**	0.07
Being taken seriously	0.39**	0.29**	0.29**	0.24**
Sufficient freedom of expression	0.32**	0.27**	0.27**	0.22**
Sufficient structure/adequate basis	0.33**	0.22**	0.26**	0.23**
Extent to which feelings could be expressed in the artwork in AT	0.72**	0.51**	0.60**	0.47**

*p < 0.05, **p < 0.01.

### Research Question 3: Patient Characteristics as Predictor

Diagnosis, age, and gender were not related to the reported benefits in general, nor to the benefits reported on daily, emotional, or social functioning; see Table 3. Only on the item “my social functioning improved thanks to art therapy” did 16.1% of the women indicate more often than men (6.1%) that their social functioning had not improved thanks to art therapy. And 18.3% of the men stated more often than the women (12.5%) that their social functioning had improved thanks to art therapy.

**TABLE 3:** Chi-square test on benefits and several variables on T2 (*N* = 528).

Items	Did you benefit from art therapy?	My daily functioning improved thanks to art therapy	My emotional functioning improved thanks to art therapy	My social functioning improved thanks to art therapy
Gender	0.72	0.12	0.84	0.03^a^
Diagnosis cluster B, C, UPD^b^	0.24	0.89	0.88	0.74

^a*^p < 0.05, ^∗^p < 0.01. ^b^Cluster B personality disorders, Cluster C personality disorders or Unspecified personality disorder.

Testing the overall model using hierarchical regression analyses, it was shown that the increase in reported overall benefits at T2 is associated with the extent to which the patient experienced being able to express his or her feelings in the artwork during the art therapy. The baseline values were predictive for increase in reported benefits of art therapy. No other specific benefit, therapeutic or patient characteristic was found to be associated with this increase. A similar pattern was shown for the effect of reported benefits on emotional functioning. Only for social functioning, the increase in reported benefits was associated with whether art therapy focused on coping with limitations and/or vulnerability. Table 4 shows the results of these hierarchical regression analyses.

**TABLE 4:** Hierarchical regression analysis between perceived benefits at T2 and variables at T1 (*N* = 528).

	Overall benefits				Daily functioning				Emotional functioning				Social functioning			

	Δ *R*^2^	*B*	SE	β	Δ *R2*	*B*	SE	β	Δ *R2*	*B*	SE	β	Δ *R2*	*B*	SE	β
Step 1: Experienced benefits on T1	0.49^∗∗^	0.52	0.07	0.54^∗∗^	0.32^∗∗^	0.45	0.07	0.44^∗∗^	0.30^∗∗^	0.39	0.07	0.39^∗∗^	0.39^∗∗^	0.49	0.07	0.50^∗∗^
Step 2: Specific benefits on T1	0.01				0.05^∗^				0.03				0.04^∗^			
Expression of emotions		0.11	0.13	0.05		0.08	0.14	0.03		0.11	0.16	0.05		0.18	0.13	0.08
Self image		–0.15	0.21	–0.07		0.07	0.13	0.03		–0.29	0.15	–0.12		–0.12	0.13	0.03
Own choices/autonomy		0.16	0.12	0.07		0.00	0.14	0.00		0.13	0.15	0.05		0.07	0.13	0.03
Recognition, insight and changing patterns		–0.03	0.11	–0.02		–0.10	0.12	–0.05		–0.03	0.14	–0.01		0.09	0.12	0.05
Dealing with limitations/vulnerability		–0.10	0.11	–0.05		–0.28	0.12	–0.14		–0.17	0.14	–0.08		–0.27	0.12	−0.13^∗^
Self-confidence		–0.02	0.12	–0.01		–0.22	0.13	–0.11		–0.13	0.15	–0.06		–0.22	0.13	–0.11
Focused attention to own bodily signals		0.16	0.10	0.08		–0.01	0.11	–0.01		0.07	0.12	0.03		–0.07	0.11	–0.03
Addressed traumatic experiences		0.13	0.11	–0.06		0.03	0.12	0.02		0.14	0.13	0.06		0.01	0.11	–0.00
Improved social functioning		0.06	0.10	0.03		–0.14	0.11	–0.07		–0.03	0.12	–0.02		0.02	0.11	0.01
Step 3: Associated factors	0.02^∗^				0.01				0.03^∗^				0.01			
Non-judgmental therapist		–0.03	0.08	–0.02		–0.04	0.09	–0.03		–0.14	0.10	–0.08		–0.05	0.08	–0.03
Being taken seriously		0.07	0.08	0.05		0.14	0.09	0.10		0.04	0.10	0.03		–0.03	0.08	–0.02
Freedom of expression		–0.05	0.07	–0.04		–0.13	0.08	–0.10		–0.01	0.08	–0.01		–0.05	0.07	–0.04
Structure/adequate basis		0.04	–0.05	0.04		0.03	–0.06	0.02		–0.01	–0.06	–0.01		0.06	–0.05	0.05
Extent to which feelings could be expressed in the artwork in AT		0.21	0.06	0.21^∗∗^		0.03	0.07	0.03		0.23	0.08	0.21^∗^		0.12	0.06	0.13^∗^
Step 4: Gender/age	0.00				0.00				0.00				0.00			
Gender		–0.01	0.10	–0.01		–0.13	0.12	–0.05		–0.12	0.13	–0.05		–0.05	0.11	–0.02
Age		–0.00	0.01	–0.04		–0.00	0.01	–0.02		–0.00	0.01	–0.03		0.00	0.01	0.03
Step 5: Diagnosis cluster	0.00	0.07	0.05	0.06	0.00	0.08	0.06	0.07	0.00	0.05	0.06	0.04	0.00	0.02	0.05	0.02

*^∗^p < 0.05, ^∗∗^p < 0.01.*

## Discussion

This study investigated the extent to which patients who have been diagnosed with a PD experienced benefits from art therapy, and whether it contributed to the improvement of their daily, emotional, and/or social functioning. It also looked at the correlation with the extent to which people can express their feelings in making artwork, with the conditions created by the therapist, and whether some persons reported more benefits from art therapy than others.

The majority of patients in the group studied (mainly Borderline, Avoidant, and PDs not otherwise specified) reported that they had gained quite a lot of benefits from art therapy in general with a mean of 3.70 (SD = 0.99) on T2 (T1: 50.3%; T2: 56.4%). The greatest positive change could be seen in answer to the overarching question as to overall benefit. Benefits in emotional functioning and social functioning also showed significant improvements, whereas any benefit to daily functioning did not involve a significant change. This fits the expectation that art therapy is focused on emotional aspects and because art therapy is mostly in a group, it also affects social functioning. The improvements are concentrated in specific target goals of which the five highest scoring goals affected were: expression of emotions, improved (more stable/positive) self-image, making own choices/autonomy, recognition of, insight in and changing of personal patterns of feelings, behaviors and thoughts and dealing with own limitations and/or vulnerability. Patients made it clear that they perceived these target areas as having been affected by art therapy and said so at both moments in time, with a higher score after 3 months.

These findings are in correspondence with previous studies in which art therapy came forward as highly suitable for and appropriate to the key problems of patients with PDs, that it offers a well-defined route to a stronger emotional awareness and contributes to constructive emotion regulation (Green et al., 1987; Franks and Whitaker, 2007; Eren et al., 2014; Haeyen et al., 2015, 2017a,b, 2018). Also, the present study looked into the interplay between the degree of benefit and various factors: the specific treatment goals, associated factors, age and gender, and PD diagnosis cluster.

A strong correlation was found between the level of benefit reported and factors such as a non-judgmental attitude on the part of the therapist, feeling that one was taken seriously, having sufficient freedom of expression, while at the same time offering sufficient structure and an adequate basis. Particularly, the extent to which patients can express their feelings in their artwork shows a strong correlation with social functioning, and is highest for overall benefit. So, when patients feel they can express themselves in the art work, they also feel they can share this experience with others. Doing so, it also affects social aspects, which fits the idea that making meaningful art in the presence of others can function as a bridge for communication about emotion related themes between the patient and others, including therapists.

Factors that did not show any significant correlation were age and gender. Art therapy in this study came forward as not primarily beneficial for men or for women, for younger or older people. Only the aspect “improved social functioning” scored somewhat higher for men than for women. This might indicate that the women see this less as a goal for themselves, whereas the group of men finds just the opposite. There was no difference between extent of benefit and the various diagnostic clusters. So in this study it made no difference whether a person had a diagnosis in the categories cluster B, cluster C, or PDs not otherwise specified. For each of these diagnostic clusters, art therapy was equally beneficial.

What ultimately came forward is that the increase in reported overall benefits is associated with the extent to which a patient has experienced that he or she can express his or her feelings in the artwork during the art therapy. No other specific benefit, therapeutic characteristic or patient characteristic was found to be associated with this increase. A similar pattern was shown for reported benefits on emotional functioning. For daily and social functioning, the increase in reported benefits was associated with coping with disability and/or vulnerability. This does not mean that only patients are indicated for art therapy who have previous experience with expressing themselves in art or have the expectation that they will be able to express their feelings in art. Being able to express feelings in art is a skill that can be stimulated in art therapy, for which coming out of their comfort zone is therapeutic and triggering this possibility for self-expression in alternative way next to verbal expression.

This study has a number of limitations. Firstly, the dropouts from art therapy were not included in the analyses since no data were known at the time of measurement 2. This may have affected the results. Even so, the sample involved was large enough to be concerned representative. A second limitation is that the period of 3 months was a period in the course of an ongoing treatment. A clearly defined starting point might yield a clearer image or clearer results. However, the beginning of a specialized treatment is seldom a clear starting point in the assistance and support programs of the patients in this target group, who have often had to deal with issues in various fields of life for many years, and have often been through numerous therapy processes, often resulting in few, temporary or variable effects. After all, their issues often consist of persistent patterns that have long existed and are typical of this diagnosis.

A third limitation is that art therapy is not isolated in this study. It was generally offered as part of a broader treatment program, so that changes in benefits might be dependent on several factors, and not just on art therapy. An argument against this is that this survey posed specific questions with a focus on art therapy. Nevertheless, this study does not have a control group so cause and effect conclusions are not possible and the repeated measures analyses yielded results with small effect sizes. A fourth limitation is that this study did not look at the relationship between the experienced benefits and aspects such as culture or IQ. These aspects might also be of influence, and it is recommended that they be included in subsequent studies. Lastly, this study gives no insight into how benefit from art therapy is achieved, what factors are operative in art therapy and what interventions are decisive in this regard. This study primarily looked at secondary factors.

The strengths of this study are that it was conducted in the target group concerned, it was close to daily practice and involved a large group of participants, meaning that a sample of sufficient size could be assumed as the starting point. As far as we know, a study of art therapy of this scope within this specific target group was not conducted previously. In addition, often the participants in this study could not specifically choose art therapy, but it was a standard part of a broader, multidisciplinary treatment plan. This does not mean that only participants with an affinity for art therapy took part, but that a considerable number of participants may have had little or no affinity with art therapy or artwork. In practice, patients often say that art therapy is outside their comfort zone. Because the study did not involve only participants with leanings toward artwork, the results would probably give a more objective picture if this had been the case.

## Conclusion

Patients diagnosed with a PD indicated that they experienced quite some benefit from art therapy. This is primarily shown in learning to express emotions, reinforcing and stabilizing their self-image, learning to make their own choices and strengthen their autonomy, recognizing, gaining insight into and changing their own patterns in feeling, thinking and acting. They also regarded learning to deal with their own limitations and/or their own vulnerability as an important goal of therapy. These target areas are perceived at the outset by patients as applicable in art therapy, and after a while it is seen even more strongly in this light.

The extent of the perceived benefits is highly dependent for patients on factors such as a non-judgmental attitude on the part of the therapist, feeling that they are taken seriously, being given sufficient freedom of expression but at the same time being offered sufficient structure and an adequate basis. Age, gender, and diagnosis cluster make no difference for the extent of the benefits experienced by a person. Art therapy provides equal advantages to a broad target group, and so this form of therapy can be broadly indicated. What does come forward is that the extent to which people perceive that they can give meaningful expression to feelings in their artwork is decisive. This provides an indication for the extent of the benefits a person can experience and can also serve as a clear guiding principle for interventions by the art therapist.

## Data Availability Statement

The datasets generated for this study are available on request to the corresponding author.

## Ethics Statement

Ethical review and approval was not required for the study on human participants in accordance with the local legislation and institutional requirements. The patients/participants provided their written informed consent to participate in this study.

## Author Contributions

SH developed the research design, conducted the research, and first authored this article. SH and SV performed the statistical analysis. SV supervised the development of the research design and research process. SV and FC co-authored this article.

## Conflict of Interest

The authors declare that the research was conducted in the absence of any commercial or financial relationships that could be construed as a potential conflict of interest.

## Footnotes

1 Art therapy is an integral part of the treatment for personality disorders in many larger national healthcare institutes in Netherlands, e.g., GGNet/Scelta, De Viersprong, Mediant, GGZ Friesland, Mondriaan, ProPersona, Synaeda, University Medical Center Groningen, GGZ Noord Holland Noord, GGZ Drenthe, GGZ Oost Brabant, Dimence and Lentis.
